# Influence of specific allergy immunotherapy (SIT) for aeroallergens on coexisting food hypersensitivity

**DOI:** 10.1186/2045-7022-3-S3-P65

**Published:** 2013-07-25

**Authors:** J Gocki, Z Bartuzi

**Affiliations:** 1 Allergy, Clinical Immunology and Internal Diseases, Collegium Medicum. University Nikolaus Copernicus, Bydgoszcz, Poland

## Background

As show our earlier study, food hypersensitivity coexist in 27,2% of patients treated SIT for aeroallergens, and more frequently occurred in patients with plant allergy – 33,3%. Coexistence of plant allergy with food hypersensitivity cold be a cross allergy, or co-allergy. In case of cross allergy, SIT for plant aeroallergens could lead to abate of food hypersensitivity. But if it is co-allergy this effect is not observe.

## Aim

The aim of this study was assessment influence of SIT for aeroallergens on food hypersensitivity outcome.

## Methods

We analysis case history 15 patients with SIT for aeroallergens and coexisting food hypersensitivity in age 32 to 72 years (medium 39 years), 11 women in age 35 to 62 years (medium 38 years), and 4 man in age 39 to 72 years (medium 44 years).

## Results

Inclusion criteria to SIT in 11 patients was allergic rhinitis (AR), in 3 patients – coexistence of asthma and AR, and in 1 patients – asthma. Oral allergy symptom (OAS) was clinical manifestation of food hypersensitivity in 12 patients. In 2 patients occurred allergic rash, and in 1 patient AR. More frequently we observed food hypersensitivity for apple – 10 patients, carrot – 7, cherry – 5, hazelnut – 4, peach – 4, nectarine – 3, celery – 3, strawberry – 2, kiwi – 2, curry – 2, pear – 2, walnut – 2, and one for: on raspberry, egg, citrus, shrimp, beetroot, morello, cow milk. Most of patients was treated SIT for trees – 6 patients, trees and grasses – 3, ragweed – 2, house dust mites – 2, grasses – 1, ragweed and trees – 1. Patient was cure SIT from 1 to 5 years (medium 2,8 years). In 10 patients we didn’t observed any changes in clinical manifestation of food hypersensitivity. Two patients observed improve of tolerance. At one patient increased symptoms. In 2 patients appear hypersensitivity for new foods. We didn`t observed food hypersensitivity outcome in none patients.

## Conclusion

In most patients SIT for aeroallergens don`t change clinical manifestation of coexistent food hypersensitivity. I some cases could lead to decreasing or increasing tolerance, or could develop hypersensitivity for new ingredients. Probably in most patients with allergy for aeroallergens, the character coexistence of food hypersensitivity is co-allergy.

## Disclosure of interest

None declared.

